# Description of the datasets for the experiments in the paper “solving the multi-vehicle multi-covering tour problem”

**DOI:** 10.1016/j.dib.2018.03.106

**Published:** 2018-03-27

**Authors:** Tuan Anh Pham, Minh Hoàng Hà, Xuan Hoai Nguyen

**Affiliations:** a Military Logistics Research Center, Military Logistics Academy, Vietnam; b GRD Department, VNG Corporation, Vietnam; c University of Engineering and Technology, VNU, Vietnam; d FPT Technology Research Institute, FPT University, Vietnam; e IT R&D Center, Hanoi University, Vietnam

## Abstract

This data article contains data related to the research article entitled, “Solving the multi-vehicle multi-covering tour problem” (Pham et al., 2017) [4]. All data of this article was generated from instances kroA100, kroB100, kroC100, kroD100, kroA200, and kroB200 from TSPLIB. It can be downloaded from public repository. This data can be used as benchmarks for the covering tour problem (CTP) variants, such as *m*-CTP-*p*, *m*-CTP, *mm*-CTP-*p*, *mm*-CTP, *mm*-CTP-o, *mm*-CTP-wo. We tested our algorithm on these data and results are shown in Pham et al. (2017) [4].

**Specifications Table**

**Table:** 

Subject area	*Computer Science*
More specific subject area	*Operational Research*
Type of data	*Numeric contained in text files*
How data was acquired	*Artificially generated*
Data format	*Raw*
Experimental factors	*Instances of six CTP variants were generated, which can be used for testing performance of algorithms on CTP problems.*
Experimental features	*We ran each algorithm 10 times for each data instance and measure average performance (accuracy) and running time*
Data source location	*Available via Internet*
Data accessibility	*All data can be downloaded from:*https://bitbucket.org/pta_vrp/mmctp/

**Value of the data**

• The data is the first problem instances for *m*-CTP problem solved in the paper.
• The data is available for download and reuse without any restriction.
• The data is stochastic and aimed for the problem of *m*-CTP and the related.

## 1 Data

This article provides datasets for six CTP variants, which are available for download from our repository (https://bitbucket.org/pta_vrp/mmctp/). These data instances can be used as benchmark data for the CTP problems [3].

## 2 Experimental design, materials and methods

### 2.1 Design

We considered six variants of the CTP problem, which are *m*-CTP, *m*-CTP-*p*, *mm*-CTP, *mm*-CTP-*p*, *mm*-CTP-*o*, *mm*-CTP-*wo*. Our objective is to investigate the performance of algorithms on these variants, especially on instances of *mm*-CTP, *mm*-CTP-*p*, *mm*-CTP-*o*, *mm*-CTP-*wo* as described in [4].

### 2.2 Materials

All these datasets were generated from instances kroA100, kroB100, kroC100, kroD100, kroA200, and kroB200 from TSPLIB (http://elib.zib.de/pub/mp-testdata/tsp/tsplib/tsplib.html).

## 3 Methods

To generate the datasets for the experiments in [4], we started with the data instances for the *m*-CTP problems given in [1], but added three more constraints to ensure that the generated data instances conform with the requirements of the *mm*-CTP problem.

• The first constraint is the maximal route length, for which we utilize the method in [2] to generate data instances that satisfy the constraint. In particular, the maximal route length *q* is computed by q=β+ρ, where $\beta =2*\max_{i\in V-\{0\}}\left(c_{0,i}\right)$ and ρ={250,500}
• The second constraint, which makes *mm*-CTP different from *m*-CTP, is that each vertex $w_{k}$ must be covered at least $u_{k}$ times. To satisfy that we generated $u_{k}$ as follows. Suppose that ${\mathrm{nb}}_{k}$ is the maximal number of nodes in *V* which can cover $w_{k}$, we randomly generated an integer $u_{k}$ in the interval from 1 to min (3, ${\mathrm{nb}}_{k}$).
• Using the graph transformation method reported in [4], we generated the data instances for *mm*-CTP-o and *mm-CTP-wo problems.*

Consequently, 192 data instances were obtained for the *mm*-CTP. Each problem data instance is labeled as *X−|T | − |V | − |W | − |p| − |q|*, where *X* is the name of the TSPLIB instance and the remaining labels are corresponding problem parameters. Some data instances do not have the *q* part indicating that the route length are relaxed for these instances.

All of this data instances can be downloaded from public repository https://bitbucket.org/pta_vrp/mmctp/. In this repository, we provided three data instance folders, namely **mmctp, mmctp_o, mmctp_wo** and with a file (**data_format.txt**) for describing the format of each data instance file. Instances in folder **mmctp** can be used for *m*-CTP-*p*, *m*-CTP, *mm*-CTP-*p*, *mm*-CTP variants.

Our algorithm was coded in C++ and ran on a 2.4-GHz Intel Xeon computer with 10 times. For more details about results, please see our paper [4].
